# P-1254. Improved Vancomycin Target Attainment Following a Quasi-Experimental Change in Pharmacokinetic Model for Bayesian Precision Dosing

**DOI:** 10.1093/ofid/ofae631.1436

**Published:** 2025-01-29

**Authors:** Maria-Stephanie Hughes, Dominic M H Tong, Jasmine Hughes

**Affiliations:** InsightRX, Boston, Massachusetts; InsightRX, Boston, Massachusetts; InsightRX, Boston, Massachusetts

## Abstract

**Background:**

When using a Bayesian approach for vancomycin dosing, pharmacokinetic (PK) models guide dosing decisions. This study explores the impact of moving from the Goti model to the Thomson model on target attainment, dosing decisions, and acute kidney injury (AKI) rates.

**Figure:**
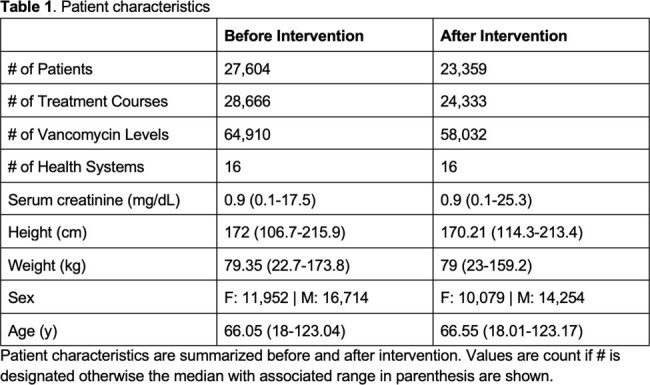

**Methods:**

Adults with BMI < 40 kg/m^2^ treated with vancomycin using InsightRX Nova Bayesian software, who switched from the Goti to the Thomson models as their default between January 1, 2023, and April 15th, 2024, were included. The switch was prompted by internal analyses suggesting the Thomson model was performing better. AUC_24_ was calculated for the treatment course and days 1, 2, and 3+. Outcomes included the proportion of patients in the target AUC_24_ range of 400-600 mg*h/L, time to target attainment, average daily dose, severe AKI rates (KDIGO stage 2 or 3 after 48 hours), and levels per course. An interrupted time series analysis was done for AUC_24_. The relationship between subtherapeutic AUC_24_ to minimum inhibitory concentration (MIC) ratio and mortality was extrapolated from a study by Lodise et al. (2014) on patients with MRSA bloodstream infections. MICs were assumed to be 1 mg/L.

**Figure:**
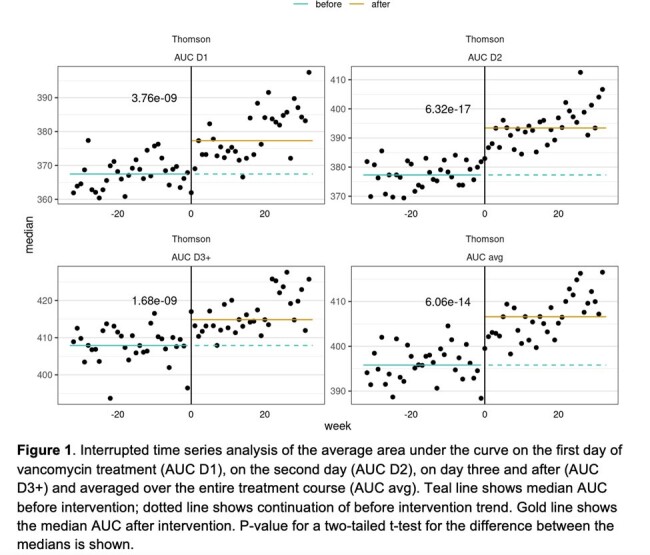

**Results:**

The study included 27,604 patients before and 23,359 after the intervention across 16 health systems (Table 1). The average AUC_24_ for days 1, 2, 3+, and the full treatment course showed statistically significant improvement (see Figure 1). AUC_24_ target attainment increased from 46.5% to 51.1% (p < 0.001) due to fewer subtherapeutic courses. Median time to target decreased from 47.8 to 28.2 hours (p < 0.001), severe AKI rates rose slightly from 1.75% to 2.03% (p = 0.023, number needed to treat (NNT): 358), and average daily dose and levels slightly increased (see Figure 2). The decreased proportion of subtherapeutic AUC_24_ on days 1 and 2 before and after intervention corresponded to expected mortality rates of 17-18% and 16-17%, respectively (NNT: 130).

**Figure:**
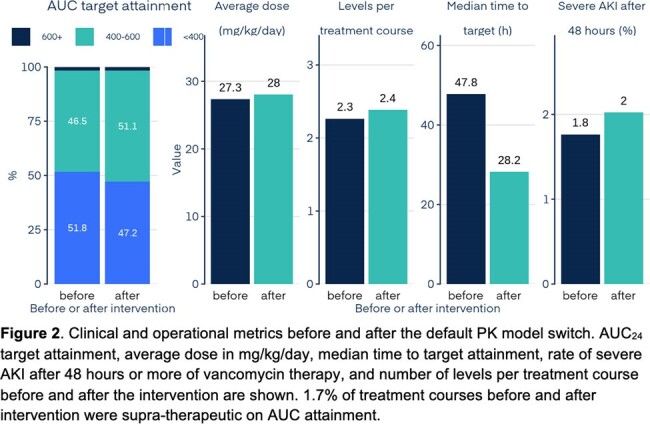

**Conclusion:**

Switching the default PK model used in a Bayesian approach for dosing adult patients on vancomycin resulted in improved AUC_24_ target attainment, and a 41% decrease in time to target attainment, with only slightly increasing AKI rates.

**Disclosures:**

**Maria-Stephanie Hughes, PharmD**, InsightRX: Employee of company|InsightRX: Stocks/Bonds (Private Company) **Dominic M.H. Tong, PhD**, Insight RX, Inc: Employee|Insight RX, Inc: Stocks/Bonds (Private Company) **Jasmine Hughes, PhD**, InsightRX: Employee|InsightRX: Stocks/Bonds (Private Company)

